# Therapeutics targeting the fibrinolytic system

**DOI:** 10.1038/s12276-020-0397-x

**Published:** 2020-03-09

**Authors:** Haili Lin, Luning Xu, Shujuan Yu, Wanjin Hong, Mingdong Huang, Peng Xu

**Affiliations:** 1 Department of Pharmacy, Sanming First Hospital, 365000 Sanming, Fujian People’s Republic of China; 2 College of Chemistry, Fuzhou University, 350116 Fuzhou, Fujian People’s Republic of China; 3 Institute of Molecular and Cell Biology, A*STAR (Agency for Science, Technology and Research), Singapore, 138673 Singapore

**Keywords:** Drug development, Proteolysis

## Abstract

The function of the fibrinolytic system was first identified to dissolve fibrin to maintain vascular patency. Connections between the fibrinolytic system and many other physiological and pathological processes have been well established. Dysregulation of the fibrinolytic system is closely associated with multiple pathological conditions, including thrombosis, inflammation, cancer progression, and neuropathies. Thus, molecules in the fibrinolytic system are potent therapeutic and diagnostic targets. This review summarizes the currently used agents targeting this system and the development of novel therapeutic strategies in experimental studies. Future directions for the development of modulators of the fibrinolytic system are also discussed.

## Fibirinolytic system: an old system as new therapeutic targets

The fibrinolytic system was originally identified to dissolve blood clots, and is shown to have important roles in other pathological processes, including cancer progression, inflammation, and thrombosis. Molecules or therapeutics targeting fibrinolytic system have been successfully used in the clinical treatments of cancer and thrombotic diseases. The clinical studies and experimental models targeting fibrinolytic system are reviewed by Haili Lin at Sanming First Hosipital, Mingdong Huang at Fuzhou University in China, and Peng Xu at A*STAR in Singapore to demonstrate fibrinolytic system as novel therapeutic targets. As an example, the inhibition of fibrinolytic system protein can be used to suppress cancer prolifieration and metastasis. This review also discusses the potential therapeutic effects of inhibitiors of fibrinolytic system on inflammatory disorders.

## Introduction

**Fig. 1:**
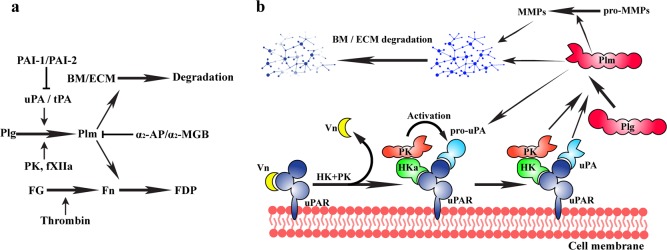
Mechanistic schemes of fibrinolytic system. **a** Scheme of the fibrinolytic system. Plasmin (Plm) mainly performs two physiological functions: 1) to degrade fibrin (Fn) to maintain vascular fluidity and 2) to degrade the ECM and BM, facilitating tissue remodeling. Plasmin is physiologically regulated by α_2_-antiplasmin (α_2_-AP) or α_2_-macroglobulin (α_2_-MGB). Plasmin is activated via the cleavage of the inactive zymogen plasminogen (Plg) by plasminogen activators (tPA/uPA) or other enzymes, e.g., plasma kallikrein (PK) and coagulation factor XIIa (fXIIa). TPA and uPA are physiologically regulated by plasminogen activator inhibitor-1 and −2 (PAI-1 and PAI-2). FG and FDP indicate fibrinogen and fibrin degradation products, respectively. **b** Kallikrein-dependent activation of the pericellular fibrinolytic system. Cells normally attach to the ECM through the binding of the D2 domain of uPA receptor (uPAR) and vitronectin (Vn). The zymogen of uPA (pro-uPA) binds to the D1 domain of uPAR. Activated high molecular weight kininogen (HKa) competitively binds to the D2 domain of uPAR to replace the uPAR-Vn interaction. HKa then recruits PK to activate pro-uPA to generate active uPA, which triggers the fibrinolytic system and facilitates the migration of cells. The generated plasmin also activates pro-uPA to amplify the activity of the fibrinolytic system.

Fibrinolysis is the process of dissolving blood clots, thereby preventing the obstruction of blood vessels. Triggered by the activation of the fibrinolytic system, fibrinolysis is mainly regulated by proteases and protease inhibitors. In addition to the originally identified thrombolytic function, the fibrinolytic system has also been found to play pivotal roles in various physiological and pathological processes, e.g., tissue remodeling, immune responses, and cancer progression. The key enzyme of this system, plasmin, has two main physiological functions: (1) to degrade the deposited fibrin into soluble fibrin degradation products (FDP) in blood vessels and (2) to degrade base membranes (BM) or extracellular matrices (ECM) to facilitate tissue remodeling or cell migration (Fig. 1a). Plasmin is physiologically modulated by a specific inhibitor, α_2_-antiplasmin, and a nonspecific protease inactivator, α_2_-macroglobulin. Plasmin is mainly derived from inactive plasminogen by tissue- or urokinase-type plasminogen activators (tPA or uPA). In addition to tPA and uPA, plasminogen can also be activated by proteases in the contact activation system: plasma kallkrein (PK) or coagulation factor XIIa (fXIIa), albeit with lower activation efficacies than those of the plasminogen activators. The activities of plasminogen activators are modulated by plasminogen activator inhibitor-1 and −2 (PAI-1 and PAI-2). As PK serves as an alternative plasminogen activator, its physiological inhibitor, C1 esterase inhibitor, is also named plasminogen activator inhibitor-3 (PAI-3).

## The Fibrinolytic System

### Plasmin and plasminogen

In blood, plasmin normally exists as plasminogen, an inactive zymogen originally synthesized in the liver and circulates at a concentration of 200 µg/mL^1^. Plasminogen is a 7-domain protein comprising an N-terminus PAN/apple domain, five tandem kringle domains and a C-terminus catalytic domain. The PAN/apple domain is proteolytically removable, and the kringle domains are responsible for fibrin binding^1,2^. The PAN/apple domain-free plasmin can be further degraded by phosphoglycerate kinase to remove the catalytic domain. The remaining five kringle domains are collectively referred to as angiostatin, an endogenous angiogenesis inhibitor^3^. Plasminogen activation by tPA is normally considered to be responsible for thrombolysis in blood vessels. In contrast, uPA-triggered plasmin generation primarily occurs in tissues and is associated with tissue remodeling^4^. Plasmin is a versatile enzyme that has a broad spectrum of physiological substrates, including many blood proteins and extracellular proteins^5^. Moreover, plasmin is the activator of multiple functional proteins. For instance, plasmin activates the generation of over 7 matrix metalloproteinases (MMPs)^6^, which directly participate in the processes of tissue remodeling, cancer progression, pregnancy, etc^7–9^. Beyond its fibrinolytic property, plasmin also has anti-coagulative effects by degrading factors V and VIII, thus blocking coagulation cascades^10^.

### UPA

UPA is a serine protease that consists of an N-terminus epidermal growth factor-like domain (GFD), a kringle domain, and a C-terminus catalytic domain. UPA is synthesized as an inactive single-chain (sc) zymogen. The activation of sc-uPA is proposed to require the binding of the membrane-bound uPA receptor (uPAR)^11^. PK, fXIIa, or plasmin sequentially approaches and activates sc-uPA^11,12^. In cancer progression, uPA is also reported to be activated by matriptase, a type II transmembrane serine protease^13^. Plasmin is the most efficient activator among these sc-uPA activators. Unlike plasmin and its myriad substrates, uPA is specific to plasminogen. However, uPA also exhibits several physiological functions independent of uPAR or plasminogen. For instance, uPAR deficiency did not affect uPA-related arterial neointima formation, neointimal cell accumulation, or smooth muscle cell migration^14^. Additionally, uPA was also reported to regulate the intravascular adherence of leukocytes independently of its proteolytic activity^15^.

### TPA

TPA, the main plasminogen activator in blood, is a serine protease containing an N-terminus fibronectin type II domain, a growth factor-like domain, two kringle domains, and a C-terminus catalytic domain^16^. Similar to uPA, tPA has no other confirmed physiological substrate except plasminogen. However, unlike uPA, tPA binds fibrin through its fibronectin type II domain and the two kringle domains. Thus, tPA specifically binds to fibrin-bound plasminogen^17^. TPA is synthesized and stored in endothelial cells as a single-chain zymogen (sc-tPA). Endothelial cells release tPA into the blood upon stimulation by thrombin, histamine, bradykinin, or other molecules. Sc-tPA is then transformed into its two-chain form (tc-tPA) by proteolytic cleavage^18^. Unlike inactive sc-uPA, sc-tPA, and tc-tPA have comparable proteolytic activity^19^. TPA is also expressed on many cells in the central and peripheral nervous systems^20–22^. Dysregulated tPA activity has been associated with multiple neurological diseases, including demyelination^23^, Alzheimer’s disease^24^, and seizures^25^.

### PAI-1

PAI-1 is the main suppressor of the fibrinolytic system and regulates tPA and uPA. PAI-1 covalently inactivates plasminogen activators and facilitates their metabolic clearance^26^. PAI-1 is synthesized in endothelial cells and stored in platelets. Once activated, platelets release PAI-1 to suppress fibrinolysis and thus promote coagulation^27^. Inflammation provokes the generation of thrombin, which also triggers the release of PAI-1 from platelets^28^. PAI-1 circulates in two forms, the active form and the latent form^29^. Active PAI-1 contains a solvent-exposed reactive central loop (RCL), which covalently blocks the active sites of plasminogen activators after proteolytic cleavage. Latent PAI-1 has an RCL that is embedded and thus loses its inhibitory activity. Active PAI-1 is unstable in vitro and irreversibly converts into the latent form within hours^30^. PAI-1 also exhibits physiological or pathological functions independent of the inhibition of plasminogen activators. For instance, as the main inhibitor of uPA, PAI-1 is expected to perform anticancer and anti-metastatic effects. However, PAI-1 was reported to manifest dual effects on the invasion of cancer cells. In different cancer cell lines, PAI-1 exhibited either suppressive^31–33^ or enhancing effects on invasion. The pro-cancer effects of PAI-1 work via the upregulation of the expression of oncogenes through other signaling pathways^34–36^.

### uPAR

uPAR is a highly glycosylated glycosylphosphatidyl-inositol-anchored receptor with three homologous domains (D1, D2, and D3)^37^. The three domains establish a pocket via an intramolecular disulfide bridge for the accommodation of the GFD domain of uPA^38^. uPAR also accommodates other ligands, including vitronectin or cleaved high molecular weight kininogen (HKa), both of which bind to the D2 and D3 domains^39,40^. Notably, uPAR has no transmembrane domain. The signal transduction caused by extracellular uPAR binding occurs via interactions between uPAR and other membrane proteins^41^, including integrins^42^, G-protein-coupled receptors (GPCR)^43,44^, and epidermal growth factor receptor (EGFR)^45^. In addition to the membrane-bound full-length form, uPAR also exists in a soluble form (suPAR) in biological fluids or as a cleaved form (cuPAR), which lacks the D1 domain^46^. After proteolytic cleavage by plasmin or uPA, cuPAR (D2-D3) loses its binding affinity for uPA or vitronectin^47,48^ and thus constitutes a negative feedback component of the fibrinolytic system. SuPAR is released from cells or plasma membranes by the cleavage of phospholipases and can exist as either intact suPAR (D1-D2-D3) or cleaved suPAR (C-suPAR, D2-D3)^49^. Intact suPAR is able to accept all extracellular proteins bound by membrane-anchored uPAR. Hence, suPAR competes with membrane-anchored uPAR in its physiological functions, such as the reduction of pericellular uPA activity and ECM binding^50^. However, the functions of uPA- and vitronectin-independent uPAR are unaffected by suPAR^51^.

## Pathological roles of the fibrinolytic system

The fibrinolytic system was first identified as a counterpart of the coagulation system. However, unlike the widely observed thrombotic diseases caused by dysregulation of the coagulation system, abnormal activation of the fibrinolytic system is clinically rare. In contrast to intravascular fibrinolysis, accumulating evidence demonstrates that extravascular fibrinolysis plays critical roles in multiple pathological processes.

### Cancer

The association between uPA-triggered fibrinolysis and cancer progression has been known for over 50 years^52^. Similar to MMPs, plasmin was originally considered to only facilitate cancer metastasis by degrading the ECM^11^. However, as its pro-cancer mechanism was revealed, scientists found that the fibrinolytic system has impacts on multiple processes involved in cancer progression. Furthermore, crosstalk with other systems has also been identified. Recent studies demonstrated that PK and HKa of the contact activation system are involved in fibrinolytic processes on the surfaces of cancer cells or immune cells (Fig. 1b). Normally, cells connect with the ECM partially through the binding of the D2 domain of uPAR on cells to the somatomedin B (SMB) domain of vitronectin in the ECM. The fibrinolytic system facilitates cancer migration through a two-step process. First, HKa binds to the D2 domain of uPAR and outcompetes uPAR-vitronectin binding. The loss of uPAR-vitronectin binding facilitates the detachment of tumor cells from the ECM. Second, HKa recruits PK or fXIIa to activate pro-uPA, which binds to the D1 domain. Active uPA sequentially activates plasminogen to generate plasmin, which further activates pro-uPA and thereby amplifies plasmin generation. The known pro-cancer functions of plasmin are (1) to release cancer-related growth factors^53^, (2) to degrade pro-apoptotic factors^54^, and (3) to promote angiogenesis^55^ either by itself or by activating pro-cancer MMPs^6^. UPA-induced fibrinolysis exhibits pro-cancer effects synergistically with transforming growth factor-β (TGF-β) and MMPs^56^. TGF-β regulates the expression of pro-MMPs and pro-uPA. As part of a feedback loop, plasmin and active MMPs activate the precursor of TGF-β, which sequentially triggers the activation of multiple pro-cancer signaling pathways in the advanced stage of cancer^57^.

### Inflammation

The fibrinolytic system exhibits critical functions involved in multiple aspects of inflammation progression. First, uPA and uPAR play pivotal roles in the extravasation of immune cells, including macrophages, leukocytes, B cells, and T cells. UPA and uPAR on the intravascular endothelial surface regulate the adherence of leukocytes to the endothelial walls^15^. Downstream plasmin and MMPs then disintegrate the BM and ECM proteins, facilitating the epithelial to mesenchymal transition (EMT) of immune cells^58^. In addition, after infection, bacteria invade and disseminate through recruitment of the host fibrinolytic system by surface-exposed plasminogen receptors to degrade the ECM or BM^59^. In addition, the fibrinolytic system has also been reported to modulate inflammation levels through the complement system. For instance, the administration of streptokinase (SK) or recombinant tPA (r-tPA) both caused activation of the complement pathway in patients with acute myocardial infarction (AMI)^60^. Similarly, both r-tPA and uPA administration caused a 2- to 3-fold increase in the plasma concentration of C3a^61^. In a septic mouse model, neutralizing uPAR suppressed the C5a signaling pathway in macrophages^62^. In glomerular mesangial cells, uPA upregulated the expression of +complement C5a receptor^63^. However, uPA-triggered plasmin generation was also reported to inactivate C5a in human fibrosarcoma cells^64^. In addition to its role in inflammation progression, plasminogen/plasmin was also reported to regulate some key steps involved in the resolution of inflammation, such as neutrophil apoptosis and macrophage reprogramming^65^.

Although no clinical data have shown that the inhibition of uPA leads to the amelioration of inflammation, numerous pieces of experimental evidence have suggested that uPA serves as a highly potent anti-inflammatory target. UPA-triggered fibrinolysis promoted the recruitment of inflammatory cells and induced organ fibrosis and dysfunction after myocardial infarction^66^. In rheumatoid arthritis (RA), fibrin deposition in synovial tissue and fluid correlates with the progression of arthritis^67^. In addition, the levels of D-dimer (a degradation product of fibrin generated by plasmin) in synovial tissue are suggested to predict the therapeutic outcomes of RA^67,68^. Plasmin facilitates the infiltration of immune cells into the synovial joints, which is impaired by knocking out plasminogen^69^. In addition, the suppression of the fibrinolytic system reduced cartilage degradation in SCID mice suffering from RA^70^. Interestingly, tPA and uPA were reported to play opposing roles in a collagen-induced arthritis mouse model; uPA^−/−^ mice showed ameliorated symptoms, while tPA^−^^/^^−^ mice showed aggravated symptoms^71^.

TPA is also upregulated in many inflammatory conditions^72^. The upregulation of tPA can be attributed to two reasons: (1) Inflammation is normally accompanied by the excessive generation of thrombin^73^, which promotes the release of tPA from storage granules in endothelial cells^74^. (2) Inflammatory cytokines, e.g., tumor necrosis factor (TNF) or interleukins (ILs), have also been reported to mediate the expression of tPA either positively or negatively^75^. For instance, TNF and IL-6 increased tPA expression in human peritoneal mesothelial cells and dental pulp cells, respectively^76,77^. In contrast, TNF and IL-1 decreased tPA levels in human umbilical vein endothelial cells (HUVEC)^75,78^. Recent evidence suggests that PAI-1 is tightly associated with inflammation, which appears irrelevant to its inhibitory effects on plasminogen activators. PAI-1 levels are locally enhanced at inflammatory sites^79,80^, which can have two causes. (1) PAI-1 stored in platelets is released upon platelet activation by excessive thrombin^28^. (2) Multiple inflammatory cytokines upregulate the expression of PAI-1^81–83^. PAI-1 deficiency significantly downregulated the acute phase levels in endotoxin-induced septic models^84,85^. In a lipopolysaccharide (LPS)-induced septic mouse model, alcohol significantly enhanced pulmonary fibrin deposition and lung injury, which was apparently ameliorated by PAI-1 deficiency^86^.

## The fibrinolytic system as therapeutic or diagnostic targets

One clinical use of plasmin inhibitor was to stop severe hemorrhage. Aprotinin, a proteinaceous plasmin inhibitor, was clinically used to prevent postsurgical or labor-induced hemorrhage^87^. However, aprotinin was withdrawn from the market in 2008 due to problems pertaining to its metabolism and specificity^88^. Although plasmin plays the most direct role in the dysregulation of the fibrinolytic system, plasmin itself is seldom considered as a therapeutic target. One likely reason is that plasmin, as a downstream factor, is involved in multiple critical biological processes, including thrombolysis and tissue remodeling. The off-target inhibition of plasmin might lead to systemic disorders. Evidence showed that plasminogen-deficient mice suffered from retarded growth, reduced fertility, and low survival^89^. In contrast, mice deficient in either tPA or uPA demonstrated slightly impaired thrombolytic properties but showed generally normal growth^90^. However, mice with both tPA and uPA deficiency demonstrated similar pathological symptoms as mice deficient in plasminogen^91^. Thus, the upstream factors, e.g., uPAR, uPA, tPA, and PAI-1, are more specific and safer targets and have several successful applications in clinical treatments.

### Therapeutics targeting uPAR

UPAR has been certified to be pivotal in multiple pathological processes and is considered to be a diagnostic and therapeutic target. Due to the overexpression of uPAR on tumor cells, pericellular uPAR is a prognostic marker for multiple cancers. In addition, as elevated uPAR levels have been connected with multiple pathological processes, uPAR agonists have also been developed for the treatment of many other diseases.

#### UPAR antagonists as anticancer or anti-inflammatory agents

UPA binds to uPAR with high affinity (*K*_*D*_ = 0.3 nM). Thus, peptides derived from the uPAR-binding regions of uPA have been studied as uPAR antagonists to interrupt uPA-uPAR interactions. Å6, an 8-mer peptide derived from uPA (residues 136–143), demonstrated excellent anticancer and anti-metastatic effects in experimental models and preclinical studies^92–94^. Å6 also entered phase I and II clinical trials for cancer treatment^95–97^. In addition, Å6 demonstrated promising therapeutic effects in other pathological experimental models. For instance, Å6 inhibited the degradation of vascular endothelial cadherin and elevated the alteration of the blood retinal barrier in diabetic rats^98^. In a laser-induced choroidal neovascularization mouse model, Å6 suppressed 95% of vascular formation^99^. In another study, Å6 inhibited retinal angiogenesis and neovascularization^100^. Moreover, Å6 suppressed LPS-induced inflammatory osteoclastogenesis and bone loss^101^. Similar to Å6, a peptide derived from residues 21–30 of uPA has also been re-engineered (21C and 29C mutants) as a cyclic peptide to inhibit uPAR. This peptide, WX-360, and its derivative, WX-360-NIe, suppressed tumor growth and metastasis in animal models^102^. Notably, the recombinant growth factor domain of uPA fused with the Fc domain of IgG suppressed tumor growth in a breast cancer–xenografted mouse model^103^ and a melanoma mouse model^104^.

Chiron Corporation screened a phage-display library to identify uPAR antagonists^105^, but no further studies were reported on the antitumor effects of these peptides. Small molecules that interrupted the interaction between uPAR and vitronectin inhibited the invasion of cancer cells in vitro^106^. The Meroueh group synthesized a series of pyrrolidinone and piperidinone derivatives that bind to the uPA-binding pocket of uPAR^107^. They also reported a series of small-molecule uPAR antagonists that orthosterically inhibited uPA-uPAR binding and simultaneously allosterically inhibited uPAR-vitronectin binding^108^. Eefting et al. reported a hybrid protein containing ATF (uPAR antagonist), TIMP-1 (MMP inhibitor), and BPTI (plasmin inhibitor) to inhibit ECM degradation in smooth muscle cells and preventvein graft diseases^109^. In addition, anti-uPAR antibodies that block the interactions of uPAR and its ligands exhibited the suppression of tumor growth and metastasis in animal models^110,111^.

#### UPAR as an antitumor target for diagnosis and therapy

Ploug et al. reported a high-affinity 10-mer linear uPAR-binding peptide, AE105 (*K*_*D*_ = 0.4 nM), which was developed by combinatorial chemistry^112^. In later studies, AE105 was conjugated with ^64^Cu-labeled DOTA, a positron emission tomography (PET) agent, for in vivo tumor imaging through binding to uPAR^113,114^. This probe showed encouraging results in its Phase I clinical trial in Denmark for the imaging of breast, prostate, and bladder cancers^115,116^. UPAR-binding ligands have also been used to promote the accumulation of antitumor agents at tumor sites. Rajagopal et al. developed a uPAR-targeting toxin by fusing a truncated Pseudomonas exotoxin with ATF, the amino terminal fragment of uPA, and evaluated the antitumor effects in vitro and in vivo^117^. In another study, full-length pro-uPA was fused with saporin to enhance its specificity for tumor cell lines^118^. In addition, ATF-conjugated iron oxide nanoparticles were developed for magnetic resonance imaging (MRI) of cancers^119,120^.

**Fig. 2:**
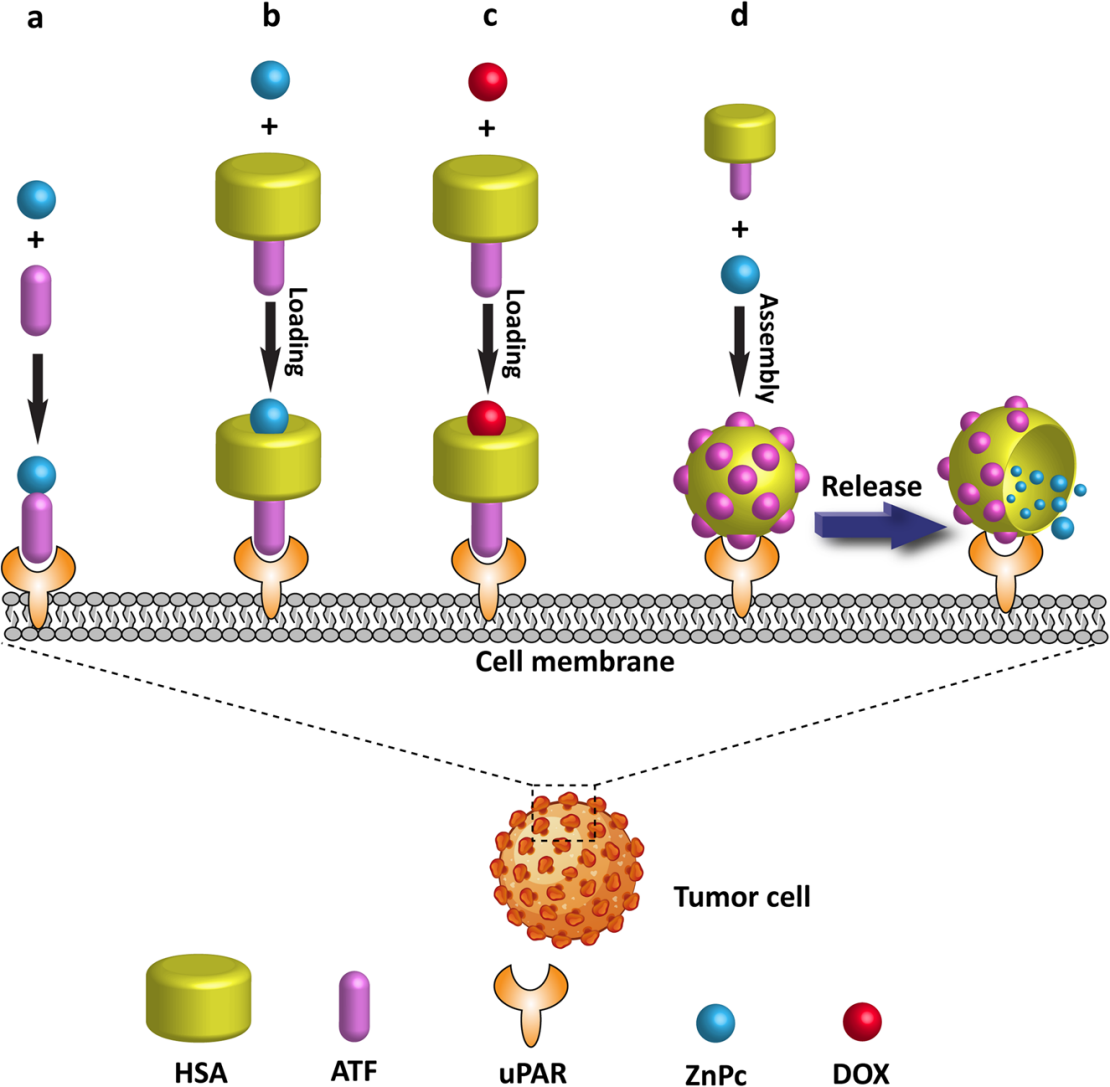
Development of therapeutics targeting uPAR. UPAR has been certified to be overexpressed on tumor cell surfaces. We developed a series of uPAR-targeting therapeutics. **a** ATF-ZnPc conjugate: a ZnPc-based photosensitizer was covalently conjugated with the uPAR-binding fragment of uPA (ATF). **b**, **c** Recombinant ATF-HSA, which integrates the uPAR-targeting property and the drug-loading property of HSA, delivered ZnPc (**b**) or doxorubicin (DOX, **c**) to tumor sites. **d** A uPAR-targeting drug carrier, developed by using ATF-HSA nanoparticles, encapsulated and released loaded ZnPc on the tumor surface.

Based on our understanding of the structural features of uPAR, we developed a series of uPAR-targeting anticancer agents (Fig. 2). First, we reported a method of uPAR-targeted photodynamic therapy (PDT) that involved the conjugation of a photosensitizer (zinc phthalocyanine, ZnPc) with ATF (Fig. 2a)^121^. Conjugation with ATF remarkably enhanced the antitumor specificity and efficacy of ZnPc in vitro and in vivo. In the following study, we developed a uPAR-targeting drug carrier, ATF-HSA, a fusion protein of ATF and human serum albumin (HSA)^122^. The fusion protein not only has the receptor binding capability of ATF but also has a long circulating time and drug carrier properties, which are derived from HSA^123^. We used ATF-HSA to deliver a ZnPc photosensitizer^122^ or doxorubicin^124^ to tumor sites in cancer-engrafted mouse models (Fig. 2b, c). ATF-HSA-loaded ZnPc was a specific tumor imaging agent and illuminated the tumor site in mice when excited at a wavelength of 630 nm. In addition to its excellent tumor-targeting capability, it also demonstrated strong antitumor effects in vitro and in vivo^122^. We also observed that ATF-HSA-loaded DOX demonstrated reduced cardiotoxicity accompanied by an enhanced antitumor effect, in contrast to free DOX^124^. In our recent work, we formulated ATF-HSA into a nanoparticle to encapsulate a larger amount of ZnPc photosensitizer (Fig. 2d)^125^. This ATF-HSA nanoparticle is able to disintegrate by contacting uPAR on the tumor surface and release ZnPc, leading to a potent photodynamic effect. Such high sensitivity to pericellular uPAR bestows the ATF-HSA nanoparticle with the property of uPAR-responsive drug release.

#### Detection of suPAR levels in plasma

Elevated suPAR levels in body fluids have been observed in multiple pathological conditions. Hayek et al. reported that elevated plasma suPAR levels were associated with decreased glomerular filtration rates and renal dysfunction in patients with chronic kidney diseases^126^. In addition, increased plasma suPAR levels were also found in patients with lung, breast, ovary, and colon cancers^127^. In addition, the plasma suPAR level was suggested as a nonspecific marker of sepsis^128^, and the determination of both the suPAR level and the procalcitonin level was able to enhance the accuracy of sepsis diagnosis^129^. Moreover, high plasma suPAR levels were also observed in patients with HIV infection^130^ or diabetes^131^. Thus, suPAR is a potent indicator of health crises^132^.

**Fig. 3:**
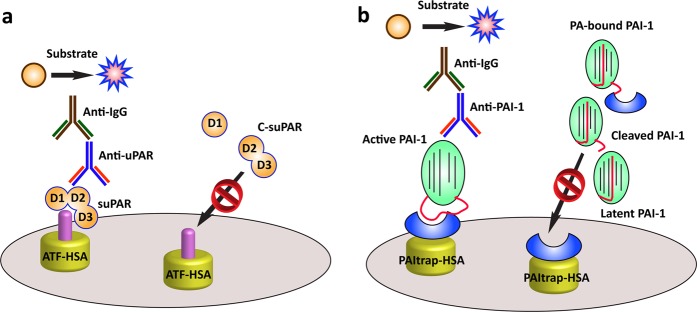
Mechanistic schemes of ELISA kits for the detection of active suPAR (**a**) and active PAI-1 (**b**).

SuPAR circulates in two forms: full-length suPAR (D1-D2-D3, also named active suPAR) and cleaved suPAR (C-suPAR, D2-D3). C-suPAR lacks the capability to bind most ligands except formyl peptide receptor-like 1 and 2 (FPRL 1 and 2)^133^. Thus, active suPAR, rather than C-suPAR, is more ideal for use as a pathological prognostic marker. Although ELISA kits have been commercially available for determining the plasma level of suPAR^134^, the detected epitope is not well defined. In addition, none of these ELISA methods can distinguish active suPAR from C-suPAR. To precisely determine the plasma level of active suPAR, we developed a new ELISA-based approach based on our recombinant ATF-HSA molecule (Fig. 3a)^135^. ATF-HSA specifically binds to the central pocket of uPAR, which accommodates uPA via its ATF fragment. The binding of uPAR to uPA requires the participation of all three D1-D2-D3 domains. Thus, C-suPAR, which lacks the D1 domain, cannot be captured by ATF-HSA. Using this approach, we found that the plasma levels of active suPAR in 20 pregnant women were significantly higher than those in healthy donors, which was consistent with the effects of the overactivation of the fibrinolytic system required for uterine remodeling^136,137^.

### UPA inhibitors as anticancer agents

In the fibrinolytic system, uPA was the first recognized target for cancer treatment. Studies of the inhibition of uPA can be dated back to the 1960s^52^. Upamostat, a prodrug of a small-molecule uPA inhibitor, was FDA-approved as an orphan drug for the treatment of pancreatic cancer in 2017. Upamostat was originally developed by WILEX AG and was licensed to RedHill Biopharma Ltd. and the Link Health Group in 2014. In its phase II clinical trial, Upamostat demonstrated only mild toxicity but enhanced the survival rates of volunteer patients^138^. Other uPA inhibitors have also been developed for antitumor uses. CJ-463 (benzylsulfonyl-D-Ser-Ser-4-amidinobenzylamide) is a 4-mer peptide-like uPA inhibitor (*K*_*i*_ = 20 nM)^139^. CJ-463 significantly suppressed cancer metastasis in a lung cancer mouse model^140^. A peptidic irreversible uPA inhibitor with an IC_50_ of 57 nM^141^ was used for the single-photon emission computed tomography of breast cancer^142^. Heinis et al. reported a series of bicyclic peptides by screening with phage display or affinity maturation^143–145^. Amiloride, an oral potassium-sparing diuretic, was found to inhibit the proteolytic function of uPA with an IC50 of 7 µM^146^. However, amiloride is not suitable for clinical anticancer treatments because of its low daily dose ceiling^147^. Buckley et al. reported an amiloride derivative with enhanced uPA affinity (*K*_*i*_ = 53 nM) and thus enhanced its antitumor effects. Meanwhile, this compound loses the diuretic effect of amiloride, suggesting that it can be used at a much higher maximum daily dose^148^. Professor Peter Andreasen of Aarhus University, in collaboration with us, reported a series of peptide-based inhibitors of human and murine uPA with high potency and specificity. The relevant studies have been thoroughly reviewed before^149^. After Professor Andreasen passed away in late 2016, we continued this project. In our recent work, we reported a highly potent and specific peptide inhibitor of murine uPA, IG-2 (*K*_*D*_ = 6.7 nM). However, in contrast to its high potency in vitro, IG-2 demonstrated moderate anticancer and anti-metastatic effects in animal models^150^, which is likely largely due to the unfavorable pharmacokinetic profile of the peptides.

### Recombinant tPA variants for the treatment of acute thrombosis

Thrombosis is caused by an imbalance in the coagulation system and the fibrinolytic system, leading to excessive fibrin deposition in blood vessels. In addition to suppressing the coagulation system, promoting the fibrinolytic system is an alternative therapeutic strategy for thrombotic diseases. Recombinant tPA (r-tPA) and purified uPA were both approved by the US FDA in 1987 and 1978, respectively, as clinical thrombolytic drugs^151^. R-tPA is more favorable than uPA as a thrombolytic agent because tPA specifically recognizes fibrin-bound plasminogen, which leads to the specific degradation of solid fibrin, while uPA does not have a plasminogen- or fibrin-binding domain and activates both circulating and fibrin-bound plasminogen, which can increase the risk of fibrinogen lysis in the blood^17,152^. R-tPA is used for the treatment of severe thrombotic diseases (e.g., acute ischemic stroke) under extremely strict administration criteria, as the administration of r-tPA confers a high risk of mass hemorrhage^153^. Several types of r-tPA have been commercially available, including Alteplase, Retaplase, or Tenecteplase, but only one uPA product is clinically used for deep venous thrombosis or pulmonary embolism^154^.

**Fig. 4:**
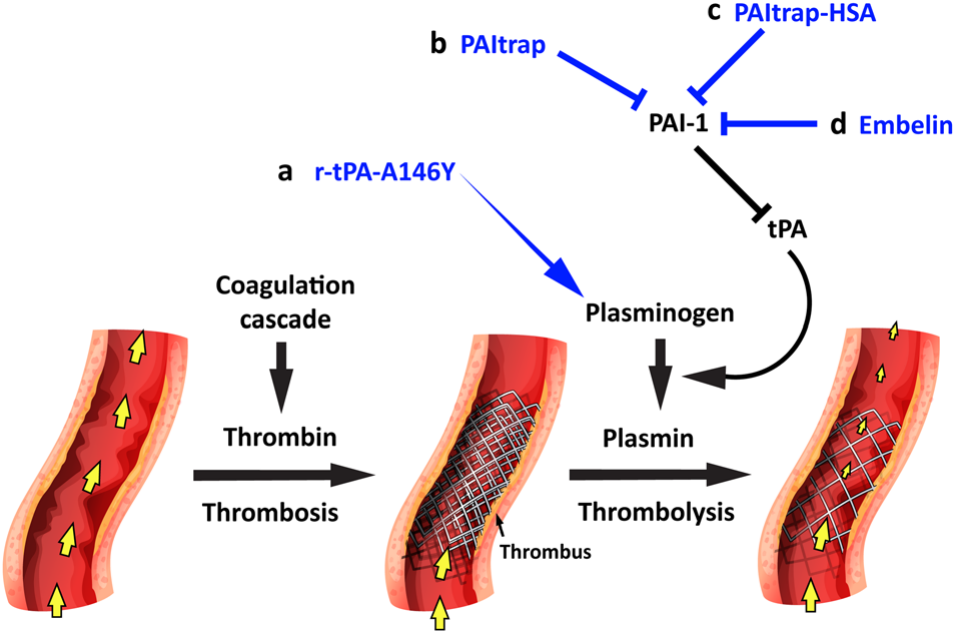
Thrombolytic agents based on r-tPA or targeting tPA or PAI-1. **a** The high-efficiency R-tPA-SPD-A146Y agent demonstrated 5-fold increased fibrinolytic activity and 30-fold increased PAI-1 resistance compared to clinical r-tPA. **b** A recombinant PAI-1 inhibitor, PAItrap, derived from the inactive catalytic domain of uPA. **c** PAItrap fused with HSA demonstrated prolonged circulation time and enhanced thrombolytic effects. **d** A small-molecule natural product, embelin, demonstrated high inhibitory potency against PAI-1 and suppressed inflammation and thrombosis in a sepsis-induced DIC model. Blood flow is indicated by the yellow arrows.

Clinically used r-tPAs require a total dose of up to 90 mg per patient to achieve sufficient thrombolytic effects. This dose is equivalent to an initial plasma concentration of ~320 nM (assuming a total blood volume of 4.5 l), which is significantly higher than the endogenous tPA concentration of ~0.1 nM. This high dose requirement is mainly due to inhibition by endogenous PAI-1 (~0.4 nM), which is further upregulated in thrombotic conditions^155^. Bennett et al. reported a full-length r-tPA variant (KtPA, with mutations of KHRR 296–299 to AAAA), which showed 90-fold increased resistance to PAI-1 and similar fibrinolytic activity as wild-type r-tPA^156,157^. In a rabbit thrombotic model, KtPA prevented fibrin deposition at lower doses than wild-type r-tPA did^158^. We also discovered an r-tPA variant with 5-fold increased fibrinolytic activity and 30-fold increased PAI-1 resistance compared to clinically used r-tPA (Fig. 4a)^159^. This new r-tPA variant was rationally designed based on our crystal structure of the tPA:PAI-1 complex^160^. In contrast to full-length KtPA with mutations in kringle domain 2, our r-tPA variant, tPA-SPD (A146Y), has only a single catalytic domain (also called the serine protease domain, SPD) with a single mutation. More importantly, our tPA-SPD variant (A146Y) enhanced fibrinolytic activity, while KtPA produced no increase in fibrinolytic activity compared to R-tPA. In a subsequent study, we used a pulmonary embolism mouse model to confirm the enhanced thrombolytic effects of tPA-SPD (A146Y) in vivo.

### PAI-1 inhibitors as thrombolytic agents

PAI-1 is the most important physiological modulator of tPA and the fibrinolytic system. Upregulated PAI-1 levels inactivate plasma tPA and suppress fibrinolysis in thrombotic or inflammatory conditions. Monoclonal antibodies against PAI-1 decreased fibrin deposition in thrombotic animal models, indicating that PAI-1 inhibition is an effective antithrombotic strategy^161,162^. Thus far, a large number of small-molecule or peptidic PAI-1 inhibitors have been developed^163,164^. Tiplasinin, also named tiplaxtinin or PAI-039, is a small-molecule PAI-1 inhibitor (IC50 = 2.7 µM) that has been widely studied in multiple experimental thrombotic models and preclinical studies^165–167^. PAI-039 docked in the vitronectin-binding region of PAI-1 in a computational model and thus inhibited vitronectin-bound PAI-1 less efficiently than free PAI-1^168^. By screening a natural product library, we identified a compound, embelin, as a potent PAI-1 inhibitor (IC50 = 1.6 µM) (Fig. 4d)^169^. Interestingly, although embelin and PAI-039 are not similar in structure, the crystal structure of PAI-1:embelin complex demonstrated that embelin also bind to the vitronectin-binding region of PAI-1. Embelin demonstrated thrombolytic effects in three thrombotic mouse models induced by electric current, FeCl_3_, and laser irradiation. By combining the fibrinolytic property we identified with the previously reported anti-inflammatory property^170^, we demonstrated that embelin is a potent therapeutic agent for sepsis-induced disseminated intravascular coagulation [to be published]. In the subsequent work, we designed and synthesized a series of embelin derivatives and found that the best compound showed a 10-fold enhanced inhibitory potency against PAI-1 (IC50 = 0.18 µM)^171^.

We also reported two proteinaceous PAI-1 inhibitors (PAItrap) derived from the inactive recombinant catalytic domain of uPA (Fig. 4b, c)^172,173^. UPA is known to bind to PAI-1 through the catalytic domain with very high affinity. We used inactive uPA (S195A) to avoid inducing proteolytic activity. Depletion of the kringle and GFD domains was to prevent off-target uPAR-binding. Based on our previously reported crystal structure of the uPA-PAI-1 complex^174^, we optimized the uPA-PAI-1 interaction by mutating some critical residues in uPA and generated a version of the catalytic domain of the uPA variant with five mutations (PAItrap), which demonstrated high affinity to PAI-1 (*K*_*D*_ = 0.15 nM). PAItrap also demonstrated high specificity among other homogenous serpin proteins, e.g., PAI-2, protease nexin 1, antithrombin, or α_2_-AP (IC_50_ > 10 µM). In a mouse thrombotic model, PAItrap significantly reduced fibrin deposition and platelet aggregation. However, as a small protein (~25 kDa), PAItrap showed a very short half-life in vivo (<5 min in mouse vessels). To enhance the circulation time, we fused PAItrap with HSA and optimized PAI-1 binding by using computational chemistry, leading to the second generation PAItrap, PAItrap(H37R)-HSA^173^. PAItrap(H37R)-HSA demonstrated an ~300-fold prolonged circulating time, a 7-fold enhanced efficacy in preventing platelet accumulation, and a 3-fold higher efficacy in reducing fibrin deposition.

### Determination of PAI-1 concentrations in plasma

PAI-1 and uPA have been validated as biomarkers for breast or prostate cancers in the clinical uses of level of evidence 1 studies by the American Cancer Society^175^. In addition, elevated plasma PAI-1 levels have also been associated with multiple pathological conditions, e.g., septic disease course^176^, hypertension in American Indians^177^, obesity adiposis^178^, and type II diabetes mellitus^179^. Thus, the determination of the plasma PAI-1 concentration is of potential diagnostic and prognostic importance. Several ELISA kits for determining plasma PAI-1 levels are commercially available. However, the results of these kits showed poor consistency, as the results determined by seven different kits varied by 4- to 6-fold^180^. One likely reason is that PAI-1 exists in four different forms, and the different kits determined the concentration of different forms of PAI-1. In ELISA assays, the target proteins are normally captured by monoclonal antibodies that typically recognize the exosites rather than the active site (RCL) of PAI-1 and thus fail to distinguish the active form of PAI-1 from other forms. We reported an ELISA assay for the precise detection of active-form PAI-1 based on PAItrap(H37R)-HSA (Fig. 3b)^181^. PAItrap(H37R)-HSA specifically recognized active PAI-1 via exposed RCL but did not bind other forms of PAI-1 in which the RCL loop was either cleaved or embedded.

## Conclusion

As a well-studied system, the fibrinolytic system has been shown to play versatile roles in many essential biological processes and is of considerable interest for the discovery of therapeutic targets. Medicinal molecules targeting the fibrinolytic system have been successfully used in clinical treatments or have been subjected to clinical trials, including the uPA inhibitor Upamostat for cancer treatment, r-tPA for acute thrombotic diseases, the radioactive uPAR-binding peptide for PET cancer diagnosis, and the plasmin inhibitor aprotinin for postsurgical hemostasis. Although these agents have achieved success in clinical treatments, their drawbacks are still obvious. For instance, Upamostat shows poor specificity, as shown by its *K*_*i*_ values that demonstrate its near-inhibition of homologous proteases, which function as coagulation factors, although no severe coagulative disorders were observed in clinical trials^182^. R-tPA is the only FDA-approved and widely used treatment for acute ischemic stroke, but it has only been used in a very small number of patients due to its various limitations. Thus, studies on the development of novel strategies with higher efficiency and specificity have shown a robust increase. This review summarizes the efforts to target the fibrinolytic system via novel diagnostic and therapeutic strategies by us and others. Accumulating evidence verifies the presence of crosstalk between the fibrinolytic system and inflammatory processes. Depletion or inhibition of uPA, PAI-1, or uPAR decreased inflammatory levels, indicating that the fibrinolytic system is a potent anti-inflammatory target. In addition, multiple studies demonstrated that the plasma levels of suPAR or PAI-1 were adequate indicators of the degree of inflammation. Medicinal modulators of the fibrinolytic system also demonstrated promising results in inflammatory disease models. Thus, modulation of the fibrinolytic system is an attractive strategy for anti-inflammatory therapies. In addition, the roles of tPA and plasmin in neuronal diseases have been increasingly recognized, strengthening the therapeutic importance of tPA. In addition, the dysregulation of PAI-1 has been associated with obesity or diabetes. Hence, the development of therapeutics targeting the fibrinolytic system is still attractive to academia and the pharmaceutical industry.
